# Dr. David C. Eisbein

**Published:** 1909-01

**Authors:** 


Dr. David C. Eisbein, of Buffalo, died at his home in this city, December 24, 1908, aged 64 years. He had been for many years a well known physician on the east side of the city, and with his wife spent a portion of last summer and autumn in European travel. He graduated at the University of Buffalo in 1878.

